# Iléus biliaire avec évacuation spontanée d’un gros calcul : a propos d’un cas

**Published:** 2010-03-11

**Authors:** Karim Ibnmajdoub Hassani, Julie Rode, Jane Poincenot, Jean-Manuel Gruss

**Affiliations:** 1 Service de chirurgie (B), CHU Hassan II Fes, Maroc,; 2 Service de chirurgie digestive, CHI Villeneuve Saint George, France

**Keywords:** iléus biliaire, calcul biliaire, Maroc

## Abstract

L’iléus biliaire est une complication rare de la lithiase biliaire; Il est caractérisé par la triade radiologique, syndrome occlusif, aérobilie et localisation ectopique d’un calcul dans le tube digestif. La cause est généralement une fistule bilio-digestive. En dehors de l’évacuation spontanée du calcul qui est rare et intéresse surtout les calculs de moins de 2 cm, le traitement reste dans la majorité des cas chirurgical. Nous rapportons le cas d’une patiente de 80ans, admise pour occlusion, chez qui le bilan étiologique retrouve la triade caractéristique de l’iléus biliaire; L’évolution a été marquée par l’émission spontanée par l’anus d’un gros calcul de 5 cm, suivie d’une nette amélioration clinique avec disparition des signes de l’occlusion.

## Introduction

L’iléus biliaire est une complication peu fréquente de la lithiase biliaire qui survient dans moins de 0,5 % des cas [1]. Il doit être soupçonné chez tout malade ayant un syndrome occlusif associé à une aérobilie et une localisation ectopique d’un calcul; cette complication doit être d’autant plus suspectée que le malade est âgé. Le plus souvent l’obstacle se situe au niveau de la région iléo-caecale, les localisations coliques sont beaucoup plus rares et représentent 2,5 % des cas [2]. Le traitement est généralement chirurgical en dehors de l’évacuation spontanée du calcul qui reste rarissime.

## Patient et observation clinique

**Figure 1:**
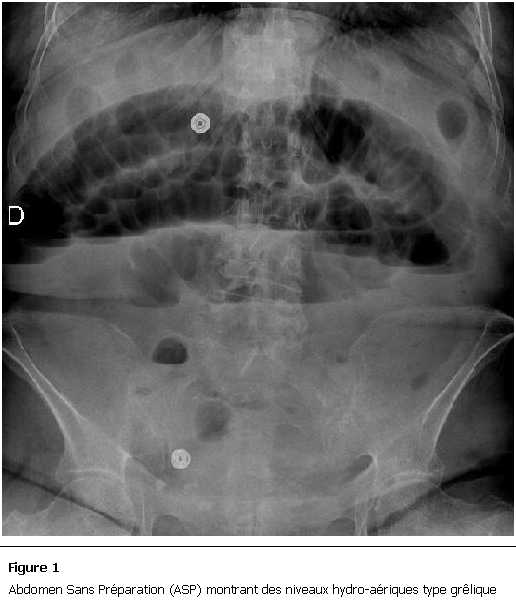
Abdomen Sans Préparation (ASP) montrant des niveaux hydro-aériques type grêlique

NP, Nous rapportons le cas d’une patiente âgée de 80 ans ayant comme antécédents pathologiques des coliques hépatiques à répétition et une hypertension artérielle, qui a présenté un syndrome sub-occlusif avec douleur abdominale, météorisme et arrêt des matières sans arrêt des gaz. Elle a été admise aux urgences chirurgicales deux jours après le début de la symptomatologie. L’examen clinique à l’admission retrouvait une patiente consciente, stable sur le plan hémodynamique (HD) et apyrétique à 37°C. L’examen abdominal retrouvait un abdomen distendu légèrement sensible et une ampoule rectale vide au touché rectal. Un bilan biologique a été réalisé et a révélé une hémoglobine à 12g/dl, une légère hyperleucocytose à 11000 éléments/mm^3^ et des plaquettes à 376 000 éléments/mm^3^. L’ionogramme sanguin était normal en dehors d’une légère insuffisance rénale d’allure fonctionnelle avec une urée à 0,78g/dl et une créatinine à 13 mg/l. Une radiographie d’abdomen sans préparation (ASP) a été réalisée et a révélée des niveaux hydro-aériques de type grêliques (Figure 1).

**Figure 2:**
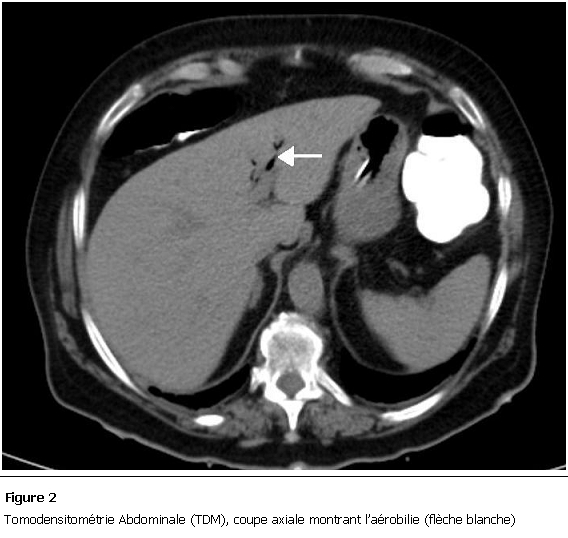
Tomodensitométrie Abdominale (TDM), coupe axiale montrant l’aérobilie (flèche blanche)

**Figure 3:**
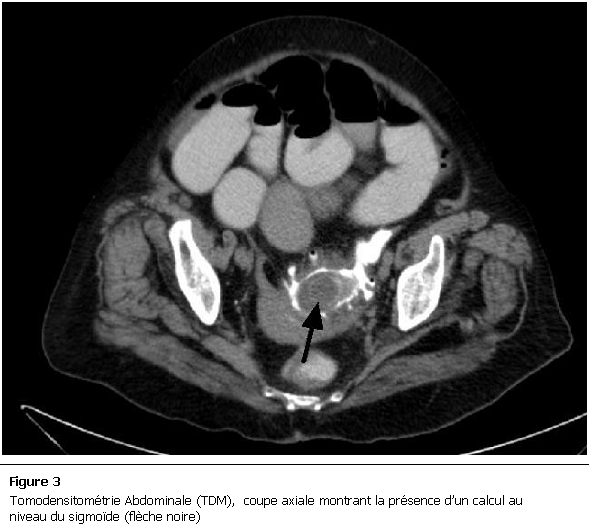
Tomodensitométrie Abdominale (TDM), coupe axiale montrant la présence d’un calcul au niveau du sigmoïde (flèche noire)

La malade a été mise en condition et une réhydratation a été débutée avec mise en place d’une sonde naso-gastrique avec compensation de la stase gastrique et surveillance de l’état HD et de la diurèse. Devant ce tableau de subocclusion inexpliquée, un scanner abdomino-pelvien a été réalisé et a objectivé des anses grêliques distendues, une aérobilie (Figure 2), et la présence d’un corps étranger de 5cm localisé dans le sigmoïde (Figure 3), le diagnostic d’iléus biliaire a donc été retenu. Devant l’absence des signes de gravité, l’amélioration de la fonction rénale quelques heures après le début des mesures de réanimation et devant la localisation distale du calcul, on a décidé de surveiller la patiente et de poursuivre le traitement médical.

**Figure 4:**
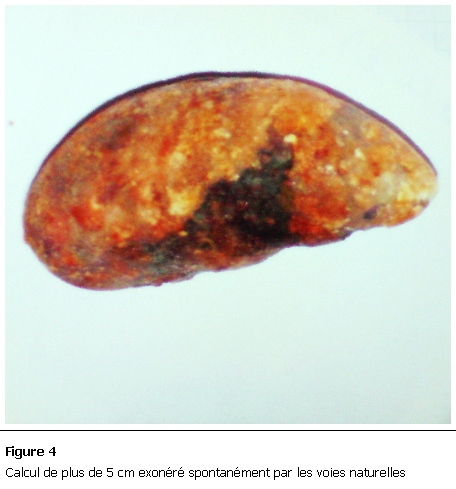
Calcul de plus de 5 cm exonéré spontanément par les voies naturelles

48h après son admission, la malade a spontanément exonéré un volumineux calcul par les voies naturelles (Figure 4). De là amélioration clinique franche avec reprise complète du transit.

## Discussion

En 1654 Bartholin a décrit pour la première fois l’iléus biliaire [3], Il s’agit d’une occlusion intestinale de type mécanique provoquée par la migration endo-luminale d’un calcul biliaire (généralement mesurant plus de 2cm), soit a cause d’une fistule bilio-digestive, soit dans les suites post opératoires d’une cholécystectomie. Ce phénomène se voit surtout chez la femme entre 70 et 80 ans, et il représente 1–3% des occlusions intestinales [4], et selon certains auteurs il constitue 25% des occlusions intestinales mécaniques Chez les personnes âgées [1]. Son diagnostic est souvent tardif, en préopératoire il se fait seulement dans 30 à 55 % des cas, devant la triade radiologique: aérobilie, syndrome occlusif intestinal de type mécanique et la localisation ectopique (souvent dans la fosse iliaque droite), d’un calcul. Cette triade attribuée à Riegler n’est observée que dans 20 % des cas [3].

L’occlusion se situe au niveau de la jonction iléo-cæcale dans la majorité des cas, la localisation colique est rare : 2,5 % des cas seulement selon la littérature [1], et le calcul est impacté généralement dans des sites où il existe une sténose inflammatoire ou tumorale, et l’élimination spontanée dans ces cas est peu fréquemment signalée [5, 6]. Dans 80 à 85 % des cas, la cause est une fistule bilio-digestive, qui dans 70 % des cas est de type cholécysto-duodénale [3], Celle-ci est secondaire à des crises répétées de cholécystite aiguë qui provoquent l’apparition de remaniements inflammatoires péri-vésiculaires.

L’iléus biliaire peut être aussi une complication de la sphinctérotomie endoscopique ou le résultat d’une dispersion lithiasique per-opératoire pendant une cholécystectomie [7]. En dehors de l’évacuation spontanée du calcul (comme c’était le cas de notre patiente), le traitement doit toujours aboutir à lever l’obstacle mécanique intestinal par entéro-lithotomie, et la réparation de la fistule a posteriori dans les meilleures conditions si la symptomatologie persistait [8]. Quoique, l’existence d’un taux élevé de complications, 5 à 46 %, chez les malades traités simplement par entero-lithotomie, pousse certains auteurs a traité en même temps la fistule [9].

## Conclusion

Les localisations coliques de l’iléus biliaire sont rares et se manifestent généralement par un syndrome occlusif. Leur traitement reste chirurgical en dehors de l’évacuation spontanée lorsque le calcul est de petite taille. C’est un diagnostic qu’on oubli souvent et auquel il faut penser devant toutes occlusion intestinale mécanique Chez une personne âgée.

## Conflits d’intérêts

Les auteurs déclarent n’avoir aucuns conflits d’intérêts.

## Contribution des auteurs

**KIM** a rédigé l’article et à contribuer à la prise des photos, **JR** a contribué à la recherche bibliographique, les deux autres auteurs ont contribué à la prise en charge thérapeutique de la malade et à la rédaction de ce document.

## Consentement

Les auteurs déclarent avoir reçu le consentement écrit du patient pour reporter ce cas.
